# Posterior Reversible Encephalopathy Syndrome With Hemorrhagic Transformation in the Postoperative Period of a Kidney Transplant

**DOI:** 10.7759/cureus.55584

**Published:** 2024-03-05

**Authors:** Edgar Dehesa-Lopez, Sergio Saul Irizar-Santana, Miguel Angel Valdez-Cruz, Sarahy Sosa-Guerrero, Fatima Lopez-Moreno

**Affiliations:** 1 Nephrology, Universidad Autonoma de Sinaloa, Culiacan, MEX; 2 Transplant, Universidad Autonoma de Sinaloa, Culiacan, MEX

**Keywords:** tacrolimus toxicity, kidney transplant recipient, convulsive seizures, severe hypertension, posterior reversible encephalopathy syndrome (pres)

## Abstract

Patients with end-stage renal disease (ESRD) who undergo kidney transplantation are at an increased risk of developing surgical and/or medical complications. Posterior reversible encephalopathy syndrome (PRES) is a rare complication that occurs in 0.34% of kidney transplant patients. It is characterized by a combination of neurological manifestations, risk factors, and characteristic radiological findings in neuroimaging studies. The development of PRES has been associated with various medical conditions and factors, including hypertension, the use of cytotoxic and immunosuppressive drugs, acute or chronic kidney disease, pre-eclampsia/eclampsia, autoimmune diseases, and solid organ and bone marrow transplantation. This report presents the case of a 19-year-old woman diagnosed with ESRD on hemodialysis due to lupus nephritis who experienced an episode of PRES with intraparenchymal hemorrhage during the postoperative period of kidney transplantation. The case emphasizes the importance of closely monitoring these patients during this period to enable early diagnosis and timely treatment of complications, ensuring a favorable prognosis.

## Introduction

Kidney transplantation is currently the most effective treatment option for patients with end-stage chronic kidney disease (ESRD) [1,2]. Proper pre-transplant assessment of the anesthetic, surgical, and immunological risk of the renal recipient has made kidney transplantation a safe procedure with a low rate of anesthetic, surgical, medical, and immunological complications. However, patients with ESRD have a higher risk of developing medical and/or surgical complications in the postoperative period due to the high prevalence of multiple comorbidities.

Posterior reversible encephalopathy syndrome (PRES) is a clinico-radiological syndrome characterized by a combination of neurological manifestations, risk factors, and characteristic radiological findings in neuroimaging studies [3]. The reported incidence in the adult population varies with rates of 98% in patients with eclampsia [4], 2.7-25% following bone marrow transplantation [5], 0.34% following renal transplantation [6], 0.84% in patients with ESRD [7], and 0.69% in patients with systemic lupus erythematosus [8].

The development of PRES has been associated with multiple factors and medical conditions, including hypertension, the use of cytotoxic and immunosuppressive drugs, acute or chronic kidney disease, pre-eclampsia/eclampsia, autoimmune diseases, and solid organ and bone marrow transplantation [3].

The purpose of this article is to report a case of PRES and emphasize the significance of postoperative medical complication surveillance in kidney transplantation for the early diagnosis and timely treatment of any complication.

## Case presentation

The patient, a 19-year-old female with blood group type B+, was diagnosed with systemic lupus erythematosus three years ago, epilepsy one month ago, and hypertension and ESRD secondary to lupus nephritis two months ago. She underwent chronic hemodialysis twice weekly for one month, showing no clinical or biochemical activity of lupus erythematosus in the past 12 months. The living related donor renal transplantation protocol was successfully completed at Hospital Angeles Culiacan in Mexico. The donor, her 45-year-old mother, also had a B+ blood type. The complement-dependent cytotoxicity (CDC) crossmatch was negative for T and B lymphocytes, and high-resolution human leukocyte antigen (HLA) typing revealed one shared HLA allele. Although the single antigen beads test was positive for DR4 (DRB1*04:01) antigen with mean fluorescence intensity (MFI) levels of 1257, it was considered a false positive result. Flow cytometry crossmatch tests were negative.

The patient experienced a generalized tonic-clonic convulsive crisis (GTCC) the day prior to the renal transplantation and was treated with intravenous diazepam and levetiracetam. She achieved complete neurological recovery following the convulsive event. The attending neurologist and rheumatologist reviewed the case and determined that the seizure event did not contraindicate the transplantation procedure. The induction immunosuppression regimen for the kidney transplant included methylprednisolone (500 mg IV) and thymoglobulin (75 mg IV). During the surgery, a complication occurred when the venous anastomosis was unintentionally unclamped, resulting in retrograde reperfusion of the graft for about 10 seconds. Despite this complication, the surgical procedure was completed successfully without further complications, including the performance of an arterial anastomosis, a venous anastomosis, and a uretero-vesical anastomosis.

In the postoperative period, the patient exhibited a urine output of three liters, hyperkalemia, metabolic acidosis, and an increase in serum creatinine (SCr) levels from 4.9 mg/dL to 6.9 mg/dL (Table 1).

**Table 1:** Laboratory characteristics of the patient H: increased values; L: decreased values

Parameters	Value	Reference values	
Creatinine (mg/dL)	6.9	0.7-1.1	H
Hemoglobin (g/dL)	9.1	11-15	L
Leucocytes (10³/µL)	9	4.5-10	
pH	7.29	7.35-7.40	L
pCO_2_ (mmHg)	35	35-45	
HCO_3_^- ^(mEq/L)	16	18-24	L
K (mg/dL)	6.3	3.5-5.5	H

A Doppler ultrasound of the kidney graft showed normal results. The rise in SCr was attributed to the episode of retrograde reperfusion during surgery; therefore, management was limited to medical monitoring, furosemide 40 mg intravenously every 12 hours, and intravenous bicarbonate at a dose of 44.5 mEq every eight hours. Over the next five days following transplantation, the patient remained normotensive, with a diuresis of 2 L/day, no signs of fluid overload, no dialysis requirement, and a progressive decrease in SCr to 3.2 mg/dL. The induction immunosuppressive regimen consisting of three doses of thymoglobulin and intravenous methylprednisolone was completed. Maintenance immunosuppression therapy was initiated, comprising mycophenolate, tacrolimus, and prednisone.

**Figure 1:**
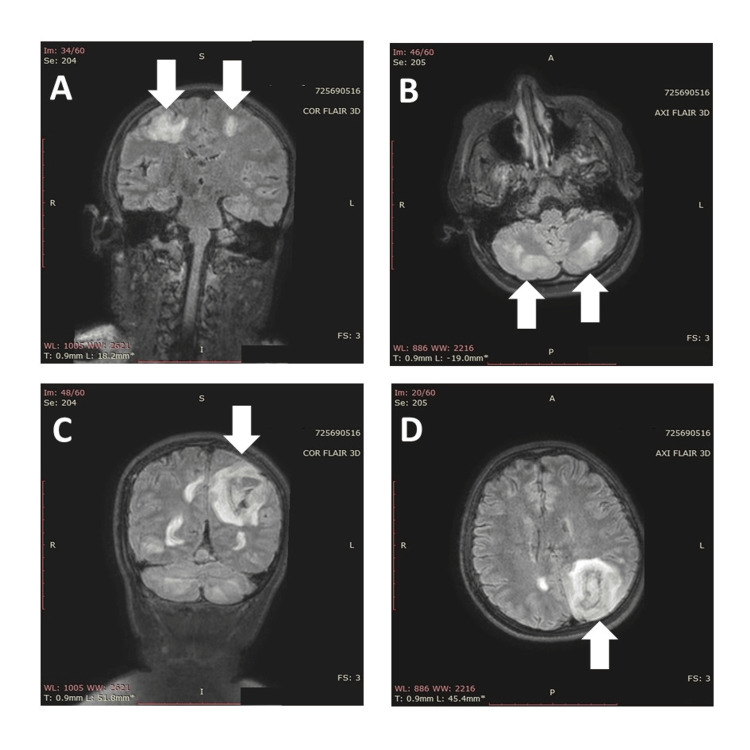
Multiple white matter hyperintense areas on FLAIR without diffusion restriction in the bilateral frontoparietal region (arrows in panel A) and both cerebellar hemispheres (arrows in panel B). Left parieto-occipital intraparenchymal hemorrhage (arrows in panels C and D) FLAIR: fluid-attenuated inversion recovery

On the seventh day post-transplant, the patient experienced a headache accompanied by sudden bilateral visual loss. Her blood pressure was recorded at 140/90 mmHg, and three hours later, she suffered a new generalized tonic-clonic seizure. During the postictal period, her blood pressure increased to 160/100 mmHg, necessitating an intravenous nitroglycerin infusion. Cranial MRI revealed multiple hyperintense areas in the white matter on fluid-attenuated inversion recovery (FLAIR) sequences in the occipital lobes, both cerebellar hemispheres, and bilateral frontoparietal regions, alongside an oval, circumscribed, and heterogeneous image on FLAIR, consistent with a left parieto-occipital intraparenchymal hemorrhage. The radiologic findings led to a diagnosis of PRES with hemorrhagic transformation (Figure 1).

The patient was admitted to the intensive care unit (ICU) for neurologic and hemodynamic monitoring. She remained hemodynamically stable with nifedipine 30 mg per os (PO) every eight hours, nebivolol 5 mg PO every 24 hours, and prazocin 2 mg PO every eight hours, in addition to an intravenous nitroglycerin infusion at 20 mcg/min. Following another seizure episode one day later, the anticonvulsant therapy was intensified with diphenylhydantoin 100 mg IV every eight hours, lacosamide 50 mg PO every 12 hours, and levetiracetam 500 mg PO every 12 hours. Due to suspected tacrolimus-associated PRES, tacrolimus was replaced with sirolimus. The serum tacrolimus level at that time was 8.7 ng/mL.

**Figure 2:**
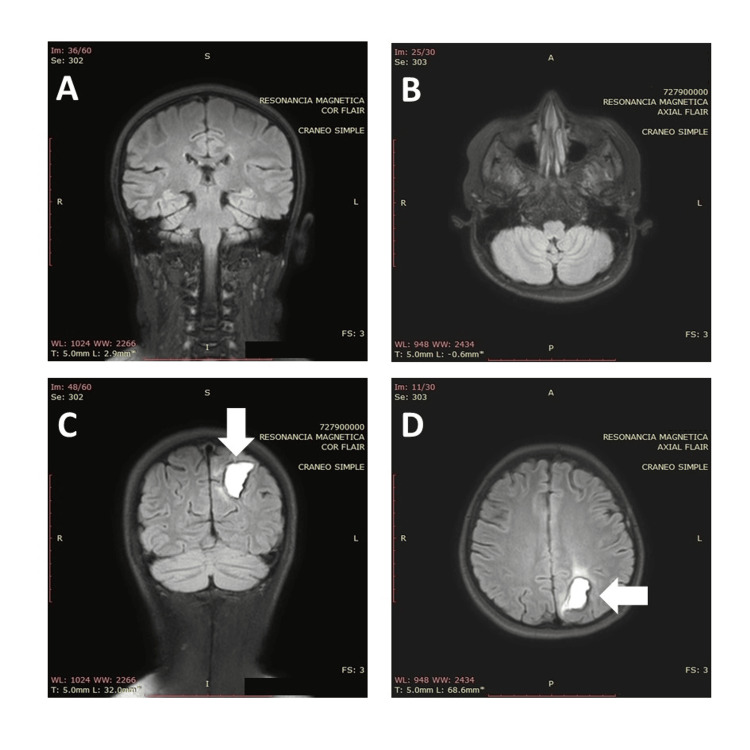
Resolution of white matter hyperintense areas in both occipital lobes, cerebellar hemispheres, and frontoparietal regions (panels A and B). Resorption data of the left parieto-occipital intraparenchymal hemorrhage (arrows in panels C and D)

The patient experienced persistent symptoms of reduced visual acuity and an ataxic gait. A follow-up cranial MRI 48 hours later indicated a reduction in vasogenic edema and no enlargement of the intraparenchymal hemorrhage. Over the next 48 hours, there was a notable progressive recovery of neurological symptoms and visual acuity, as well as improved blood pressure management, enabling a gradual discontinuation of the intravenous nitroglycerin infusion. From the renal point of view, a progressive decrease in SCr to 1.7 mg/dL was noted, and the patient was discharged 14 days after the transplantation. Control MRI showed resolution of vasogenic edema and reabsorption of intraparenchymal hemorrhage (Figure 2). Five months following the PRES episode, the patient remains asymptomatic with full visual and neurological recovery, no further seizure episodes, an SCr of 1.1 mg/dL, and ongoing treatment with mycophenolate, sirolimus, and prednisone.

## Discussion

PRES is a neurological disorder characterized by a range of symptoms including headache, mental status changes, visual disturbances, seizures, and loss of consciousness. These clinical manifestations are accompanied by vasogenic edema affecting the posterior regions of the cerebral circulation, as observed in neuroimaging studies. The precise incidence of PRES remains undetermined, and while it has been reported across all age groups, the mean age of presentation is approximately 45 years [3]. It is more common in women, even after excluding cases of PRES associated with eclampsia. PRES has been observed in various patient populations, with a notable incidence in up to 98% of patients with eclampsia [4]. In the context of bone marrow transplantation, PRES incidence ranges from 2.7% to 25% [3,5]. Among solid organ transplant recipients, the reported incidence varies between 0.4% and 6% [6]. Additionally, PRES occurs in 0.4-0.8% of patients diagnosed with either ESRD [7] or systemic lupus erythematosus [8].

The causes of PRES are multifaceted, encompassing a range of medical conditions and pharmacological agents. Notably, PRES has been linked to hypertension, pre-eclampsia/eclampsia, sepsis, acute and chronic kidney injury, as well as bone marrow and solid organ transplantation. Additionally, the administration of various immunosuppressive medications, antibiotics, and cytotoxic drugs has also been implicated in its development (Table 2) [3].

**Table 2:** Conditions associated with the development of posterior reversible encephalopathy syndrome Triplett et al. [3]

General conditions	Cytotoxic and immunosuppressive medications
Hypertension	Hydroxydaunorubicin/adriamycin
Sepsis	Vinblastine/vincristine
Solid organ transplantation	Gemcitabine
Eclampsia and pre-eclampsia	Cisplatin, oxaliplatin, and carboplatin
Renal failure	Bortezomib
Malignancy (solid organ and hematological)	Cyclophosphamide
Bone marrow transplantation	Daunorubicin
Stem cell transplantation	Interferon therapy
Hypomagnesaemia	Capecitabine, 5-fluorouracil
Hypercalcaemia	Cytarabine
Hypercholesterolaemia	Etoposide
Late radiation-associated encephalopathy	Corticosteroids
Autoimmune disorders	Rituximab
Rheumatoid arthritis	Ciclosporin
Crohn’s disease	Tacrolimus
Systemic lupus erythematosus	Sirolimus
Scleroderma	Mycophenolate mofetil
Vasculitis	Methotrexate
Neuromyelitis spectrum disorder	Azathioprine
Toxins	Other medications
Scorpion poison	Lithium
Lysergic acid diethylamide (LSD) intoxication	Linezolid
Ephedra overdose	Intravenous contrast
Alcohol intoxication	Intravenous immunoglobulin
Cocaine	Tyrosine kinase inhibitors

The clinical manifestation of PRES encompasses a spectrum of neurological symptoms. Headaches, reported in approximately 50% of patients, typically present as diffuse and gradually onset. Encephalopathy, observed in 28-94% of cases, varies from confusion and mild cognitive impairments to more severe conditions such as stupor and coma. Seizures, a common symptom occurring in 74-87% of individuals, usually manifest within the initial 24-48 hours following symptom onset. In rare instances, seizures serve as the initial symptom, with 3-17% of cases escalating to status epilepticus [3].

Visual disturbances are noted in 39% of patients, displaying a wide range from diminished visual acuity and diplopia to more complex symptoms like changes in visual fields, alteration in color vision, visual hallucinations, and even cortical blindness [9].

The pathophysiology of PRES remains a subject of debate with several complementary theories proposed to explain its underlying mechanisms. The vasodilation/hyperperfusion theory postulates that the acute development of arterial hypertension causes a loss of autoregulation of cerebral blood flow, resulting in vasodilation, hyperperfusion, and vasogenic edema. This theory would explain the more frequent involvement of posterior brain regions where the capacity for flow autoregulation is lower and is supported by the fact of rapid clinical and radiological improvement with urgent treatment of hypertension. However, it would not explain the cases of PRES in normotensive patients, such as those observed in solid organ transplant patients or associated with the use of immunosuppressants [10]. Alternatively, the vasoconstriction/hypoperfusion theory posits that the release of potent vasoconstrictors, specifically endothelin-1, prostacyclin, and thromboxane A2, triggered by endothelial damage from infections, sepsis, the use of immunosuppressive drugs, and autoimmune disorders leads to vasospasm, ischemia, and subsequent cerebral edema. This hypothesis offers an explanation for the occurrence of PRES in patients without hypertension, highlighting the intricate role of endothelial dysfunction in the syndrome's pathogenesis [10-12].

Neuroimaging studies play a pivotal role in the confirmation of PRES diagnosis, elucidation of the extent and severity of cerebral involvement, and identification of potential concurrent complications. Although vasogenic edema may be seen on non-contrast CT in some patients, MRI of the brain is the gold standard due to its superior resolution, especially of posterior fossa structures [3]. The hallmark radiological patterns of PRES include subcortical, bilateral, and symmetric vasogenic edema predominantly in the parieto-occipital regions. These features are most discernible in FLAIR MRI sequences, appearing in up to 70% of cases. The three anatomical patterns most frequently identified in neuroimaging are the parieto-occipital pattern, the holohemispheric watershed pattern, and the superior frontal sulcus pattern [13].

Hemorrhagic complications, such as intraparenchymal or subarachnoid hemorrhage, may occur in 10-25% of patients with PRES [3]. In their retrospective study of 151 patients diagnosed with PRES, Hefzy et al. reported an incidence of hemorrhagic complications of 15.2%. These were more common in transplant patients treated with immunosuppressants (22%) and those with immunologic disorders (16.7%) and less common in patients with PRES associated with eclampsia (5.6%) [14].

The definitive diagnosis of PRES is established through an integrative approach, encompassing the neurological presentation, radiological evidence of vasogenic cerebral edema from neuroimaging studies, and the identification of associated risk factors. These factors include an abrupt increase in blood pressure, renal failure, pre-eclampsia/eclampsia, autoimmune diseases, infections, recent transplantation, and the administration of immunosuppressive and chemotherapeutic agents. This comprehensive diagnostic methodology ensures the accurate identification of PRES, facilitating appropriate management and intervention.

The cornerstone of acute management for PRES lies in the prompt recognition and diagnosis of this complication. There are no specific treatment guidelines for this type of patient, and in general, treatment is aimed at providing supportive measures and reversing or eliminating any condition or factor associated with the risk of developing PRES. In the case presented, medical management concentrated on rigorous blood pressure control, seizure management, and the discontinuation of tacrolimus, identified as a probable precipitating agent.

In the management of hypertension within the context of PRES, specific guidelines remain undefined. For patients experiencing acute arterial hypertension, it is imperative to approach blood pressure reduction cautiously. A gradual decrease, not exceeding 20-25% of the initial value within the initial hours, is advised to prevent the potential risks of cerebral, coronary, and renal ischemia. The target mean arterial pressure (MAP) should ideally be maintained within the range of 105-125 mmHg. To mitigate blood pressure variability and ensure more consistent control, the administration of intravenous antihypertensive medications is recommended. First-line intravenous antihypertensive agents for the management of hypertension in patients with PRES include nicardipine, administered at dosages ranging from 5 to 15 mg/hr, labetalol at 2 to 3 mg/hr, and nimodipine at 0.5 to 1 mg/hr. Second-line antihypertensive agents encompass sodium nitroprusside, hydralazine, and diazoxide [3]. The use of intravenous nitroglycerin in patients with PRES and hypertension is controversial because of reported cases of worsening cerebral edema following its administration [15]. However, in our case, we decided to use IV nitroglycerin because it was the only intravenous antihypertensive available in our hospital, and the controversy over its use in these patients is based on observations from case reports without conclusive evidence of a cause-and-effect relationship. The final recommendation for the use of intravenous nitroglycerin in patients with PRES and hypertension is to maintain close neurological monitoring after initiation of nitroglycerin and to discontinue the medication immediately if neurological deterioration occurs.

In the absence of specific guidelines for the management of seizures in patients with PRES, treatment strategies necessitate a tailored approach. The antiepileptic drugs most frequently administered to hospitalized patients include benzodiazepines, levetiracetam, and phenytoin. It is noteworthy that the development of epilepsy as a sequelae of seizures associated with PRES is relatively rare, occurring in only 1.0-3.9% of patients [16]. 

Among transplant recipients undergoing immunosuppression with tacrolimus, a significant proportion, ranging from 40% to 60%, may encounter mild neurotoxic effects, including symptoms such as headache, paresthesias, tremors, sleep disturbances, and dysesthesias. However, a smaller fraction, up to 1.6%, could experience more severe neurological adverse effects. These include psychosis, hallucinations, cortical blindness, seizures, cerebellar ataxia, and PRES [17]. The primary recommendation for managing severe neurotoxicity attributed to tacrolimus involves the discontinuation of this medication. Subsequently, a transition to an alternative immunosuppressant, such as cyclosporine or sirolimus, is advised [18].

In the case presented, the patient necessitated admission to the ICU for neurological and hemodynamic monitoring. Approximately 70% of patients diagnosed with PRES require ICU admission, underscoring the critical nature of this syndrome and the need for intensive management. The primary indications for ICU transfer include the manifestation of encephalopathy, the occurrence of seizures, respiratory depression, and the requirement for invasive management of arterial hypertension. Furthermore, a significant portion of these patients, approximately 35-40%, may require mechanical ventilation, with the duration of ventilation typically ranging from three to seven days [3].

The prognosis of PRES depends mainly on its underlying causes. Despite its characterization as a generally reversible condition with favorable clinical outcomes, data indicate a mortality rate of approximately 19% and the occurrence of functional sequelae in up to 44% of patients [3,19]. Follow-up imaging studies have documented the presence of residual structural lesions in up to 40% of cases [20]. On the other hand, Hefzy et al. reported in their retrospective study that 70% of patients with PRES and hemorrhagic complications had a complete neurological recovery, with a mean recovery time of 6.6 days (range, 2-12 days) and a hospital stay of 15 days (range, 3-76 days). This data suggests that, despite the potential for severe complications and prolonged hospital stays, a substantial proportion of patients with PRES, even those with additional complications such as hemorrhagic complications, can expect significant recovery [14]. 

## Conclusions

Patients with ESRD who undergo kidney transplantation are at increased risk of developing any type of surgical and/or medical complication. PRES with hemorrhagic transformation is a rare but critical complication in the postoperative period after kidney transplantation. Close monitoring of these patients during this period is of paramount importance for early diagnosis and timely treatment to ensure a favorable prognosis for the patients.
